# Variable Food Begging Calls Are Harbingers of Vocal Learning

**DOI:** 10.1371/journal.pone.0005929

**Published:** 2009-06-16

**Authors:** Wan-chun Liu, Kazuhiro Wada, Fernando Nottebohm

**Affiliations:** 1 Laboratory of Animal Behavior, The Rockefeller University, New York, New York, United States of America; 2 Division of Integrated Life Science, Hokkaido University, Sapporo, Hokkaido, Japan; University of Sussex, United Kingdom

## Abstract

Vocal learning has evolved in only a few groups of mammals and birds. The developmental and evolutionary origins of vocal learning remain unclear. The imitation of a memorized sound is a clear example of vocal learning, but is that when vocal learning starts? Here we use an ontogenetic approach to examine how vocal learning emerges in a songbird, the chipping sparrow. The first vocalizations of songbirds, food begging calls, were thought to be innate, and vocal learning emerges later during subsong, a behavior reminiscent of infant babbling. Here we report that the food begging calls of male sparrows show several characteristics associated with learned song: male begging calls are highly variable between individuals and are altered by deafening; the production of food begging calls induces *c-fos* expression in a forebrain motor nucleus, RA, that is involved with the production of learned song. Electrolytic lesions of RA significantly reduce the variability of male calls. The male begging calls are subsequently incorporated into subsong, which in turn transitions into recognizable attempts at vocal imitation. Females do not sing and their begging calls are not affected by deafening or RA lesion. Our results suggest that, in chipping sparrows, intact hearing can influence the quality of male begging calls, auditory-sensitive vocal variability during food begging calls is the first step in a modification of vocal output that eventually culminates with vocal imitation.

## Introduction

Vocal learning has evolved in a few groups of birds and mammals [1], [2]. It remains unclear how and why vocal learning has evolved and particularly how brain circuitries that produce an innate vocal repertoire were modified to enable vocal learning. Vocal learning develops in early life in altricial young while their postnatal brains are growing rapidly. One may suspect that early vocal experience influences development and evolution of vocal learning [3]. Here we use an ontogenetic approach to examine how and when vocal learning starts and what is being learned.

Peter Marler [4] characterized vocal learning as “the development of a vocal pattern that requires intact hearing”. He was mindful that the vocalizations of domestic fowl, doves, and suboscines show little variability among individuals and are normal even after early loss of hearing [5]–[7]. In stark contrast, the vocalizations of songbirds, parrots, and some hummingbirds, require for their normal ontogeny intact hearing and access to external models that are imitated [8]. Marler was aware that this separation between hearing-dependent and non-hearing-dependent vocal ontogeny is not restricted to imitation. Oregon juncos, *Junco oreganus*, are able to imitate external models but can also produce songs they have not heard before. When hand-reared in groups they develop larger song repertoires than when reared singly and this increase in repertoire size results not from individuals copying each other, but from “vocal improvisation” [9], [10]. In a follow-up study, Konishi [11] showed that when juncos were deafened before the onset of song, the quality of their song differed considerable from that of the birds just reared in isolation. Clearly, hearing can influence song development even in the absence of an external model. Kroodsma [12] has further remarked that large, improvised song repertoires occur also in other songbirds, such as catbirds, *Dumetella carolinensis* [13], and sedge wrens, *Cistothorus platensis* [14], whose close relatives are otherwise known for their very numerous and accurate vocal imitations

Hearing could modify the vocal output of a bird that is not imitating a model in at least four ways: 1) An innate filter or template that focused on auditory feedback from the bird's own developing vocalizations could encourage the production of some sounds but reject others [8], [15]. A reference system of this kind is likely to be in place since all males and females must respond appropriately to conspecific songs they have not heard before and that, in the case of females, they are not able to produce. 2) Konishi [8] was aware that the guidance provided by innate template would be hard to distinguish from a developing vocal-motor program based on the progression of a “fixed input-output relationship”, where input refers to auditory feedback. 3) Early stages in vocal ontogeny could map the acoustic space of the bird's vocal organ, teaching a young bird the acoustic consequences of various vocal gestures. Along these lines, Thorpe and Pilcher [16] suggested that the subsong of birds and babbling of infants could be thought of as a form of experimentation or play, a way to generate vocal experience that could be later applied to the imitation of external models. 4) Auditory feedback could also act as a stimulus for the unfolding and expression of latent programs that in themselves are not learned. We know that in songbirds a same pathway and even a same set of cells can respond to sound and also be active in the production of sounds [17]–. Vocal pathway neurons could respond to sound by releasing trophic substances that promoted local circuit growth in a manner that affected vocal output. These four mechanisms need not be mutually exclusive and they would all be interrupted by deafening. Their action could give rise to the behavior that Marler et al. [9] referred to as “improvisation”. But even with these caveats, the fact remains that hearing dependent “improvisation” is known to occur only in bird species that show vocal imitation, suggesting that these two behaviors share underlying mechanisms.

Here we study the early development of vocal learning and its circuitry in a songbird, the chipping sparrow, by examining major characteristic of vocal learning: its dependence on auditory feedback, a protracted vocal ontogeny [16], [20], and its association with a specialized forebrain song system [21]; these features are absent in vocal non-learners [5]–[7], [22]–[24]. Our results reveal that the first vocalizations of male chipping sparrows, the food begging calls, show features that are associated with the production of learned sounds.

## Results

**Figure 1:**
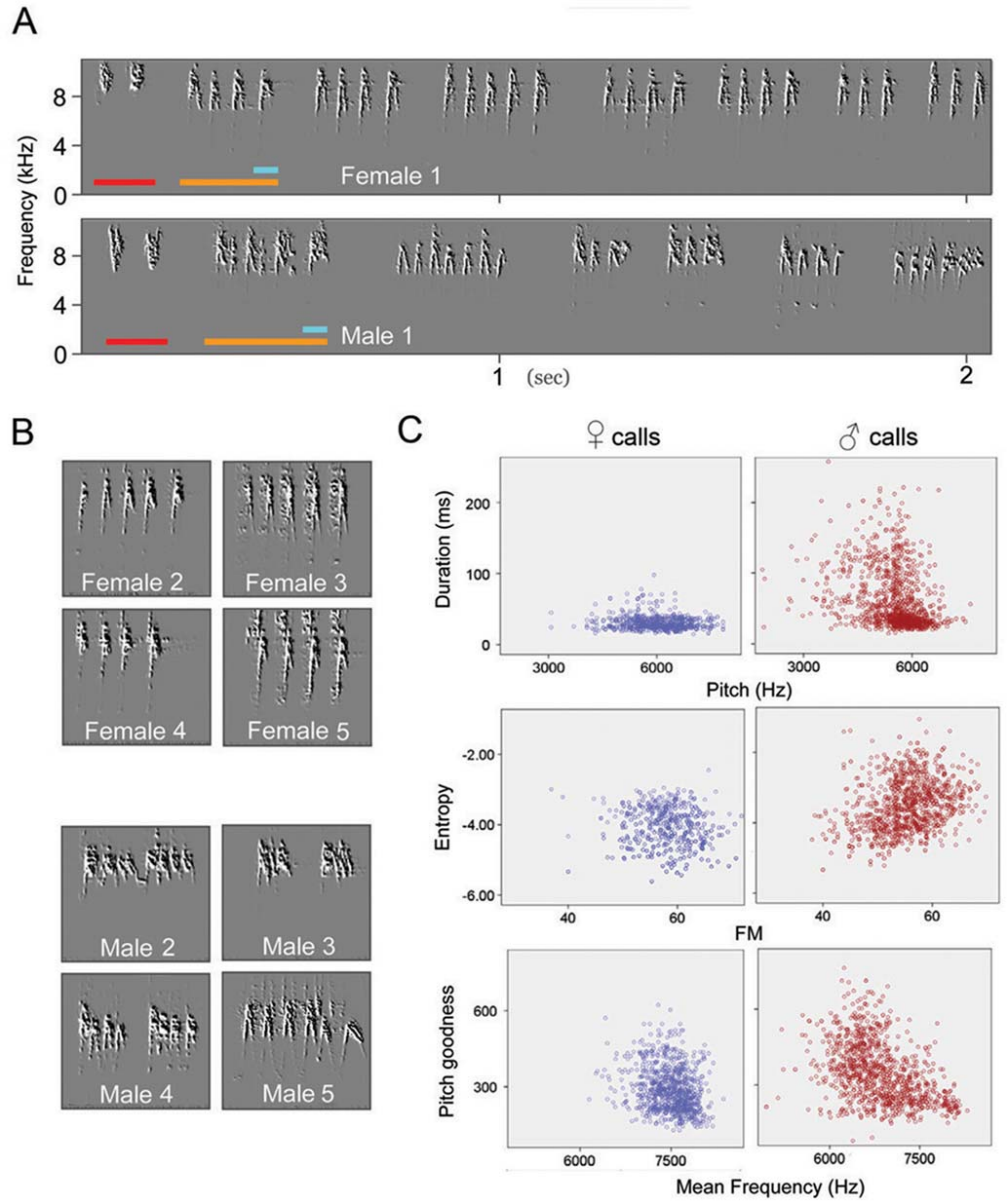
Sexual dimorphism of food begging calls. (A) The food begging calls of the females are more stereotyped than those of males at PHD20. Each call note (light-blue bar) is repeated 3–7 times in a rendition (orange bar). Prior to begging calls, juveniles produce “chip” contact calls (red bars) as a parent approaches. (B) The begging calls of each female shown came from a different clutch (females 2–5), but males 2–5 are siblings from the same clutch at PHD 20. (C) Higher call variability in juvenile males (n = 13) than females (n = 12) at PHD 20 is seen as the scatter plot distribution of entries for six acoustic features: duration, pitch, Wiener entropy, frequency modulation (FM), pitch goodness, and mean frequency. Male calls were significantly different from female calls in these features (see Fig. S2). Each dot represents a female (blue) or male (red) call note.

The begging calls of chipping sparrows became audible at post-hatching day (PHD) 3–5. Most juveniles reached independence and stopped begging at PHD 30–36. We define food begging calls as the vocalizations produced by a juvenile when food is presented a few inches in front of it (Movies S1, S2). Initially, the food begging calls were high-pitched pure tones (Fig. S1). After fledging (PHD 9–11), two different call types emerged: the food begging calls of fledglings and the “chip” contact call (Fig. 1A). Each individual bird produced a single type of food begging call, though the calling intensity (i.e., the number of repeated notes per food-begging bout), the calling rate (number of call renditions per unit of time), and amplitude varied with the degree of hunger. “Chip” Contact calls were emitted prior to the food begging calls as parents approached. This contact call is functionally and morphologically similar to the contact call of adults.

The food-begging calls differ between males and females (Figs 1, S2). This difference first became apparent around PHD11∼14, soon after juveniles fledged. The begging calls of males were more variable than those of females (n = 13 males, 12 females; 300 call notes per bird at PHD 15 and 25; MANOVA with 6 sound features; Wilk's Lamda = 0.63, F = 56.1; P<0.025 at PHD 15; F = 80.7; P<0.001 at PHD 25; Audios S1,S2). Male calls were also significantly different from those of female calls in several acoustic features (Fig. S2). By contrast, the calls of young females were rather stereotyped and differed little between individuals (Fig. 1). We did not find sexual differences of the “chip” calls (n = 6 males, 6 females; 50 notes each at PHD 25; Wilk's Lamda = 0.187; F = 19.5; P>0.1).

**Figure 2:**
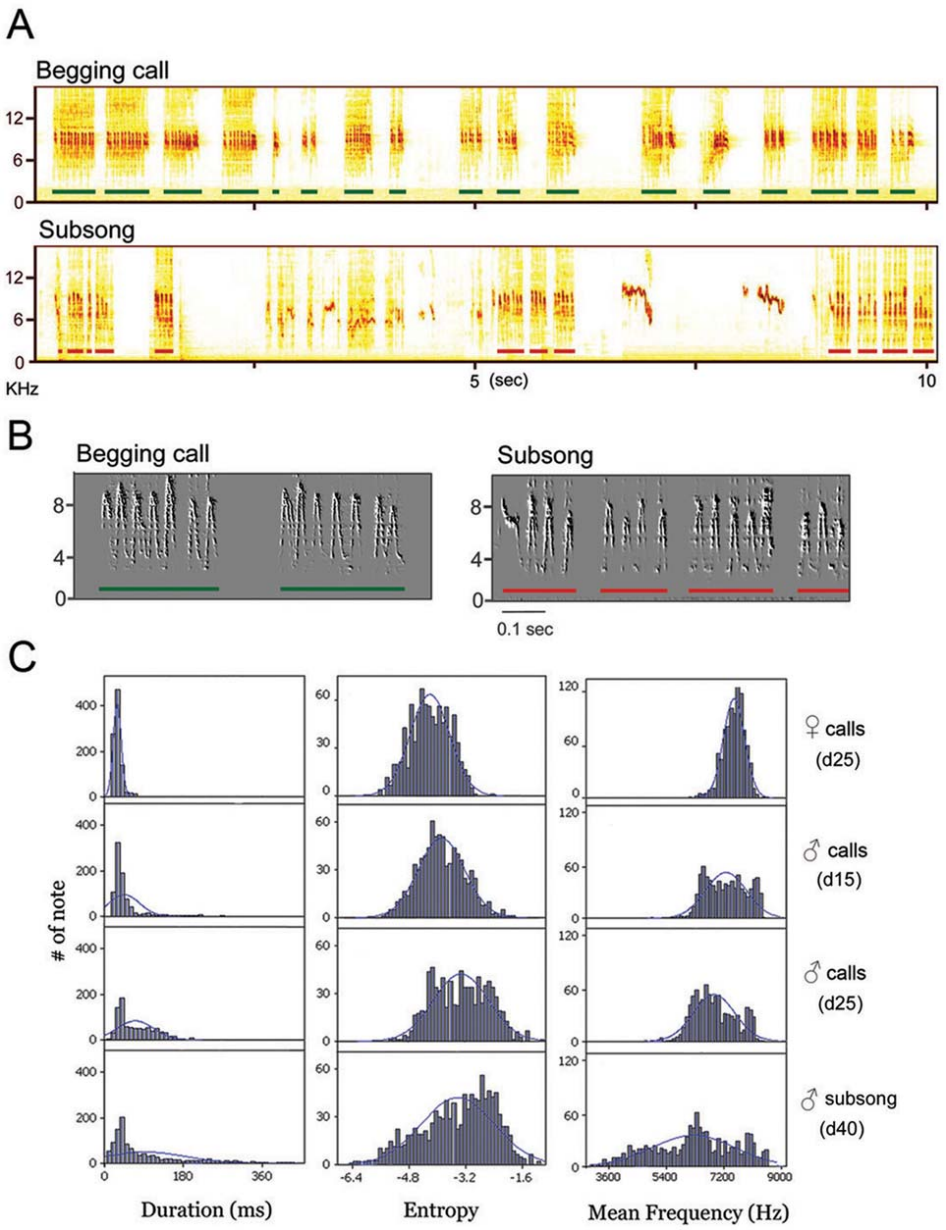
Close resemblance between food begging calls and early subsong. (A) Food begging bouts (green bars) produced by a juvenile male at PHD25 and similar sounds in that bird's early subsong (red bars) at PHD 39.(B) A closer view of late begging calls and early subsong from the same male. (C). Three acoustic features (mean duration, Wiener entropy, mean frequency) of early subsong (n = 13 males at PHD 40) are more similar to those of late begging calls of males (n = 13; MANOVA, Wilk's Lamda = 0.086, P>0.05; Tukey post-hoc test)) than to those of females (n = 12) (80–85 call and subsong notes per bird) at PHD 25 (see Fig. S3 for detailed analysis).

The food begging calls of juvenile males closely resembled some of the sounds from early subsong, though the behavioral context was very different. Food begging stopped around PHD 30–36, and subsong was first recorded around PHD 28–40. Some males (2 of 13) started to produce subsong before they stopped food begging. Early subsong occurred when young males were well fed and, with their feathers fluffed and eyes closed, seemed to nap during the daytime (Movie S3). Unlike food begging, this subsong behavior was not directed at another individual. Subsong was much softer (mean amplitude = 31.6±3.5 dB) than begging calls (n = 5 males; 62.1±5.7 dB; n = 300 notes each) and showed greater variability in note structure (Fig. 2A). Some of the sounds of early subsong were very reminiscent of late begging calls in males (Audios S3, S4). This close similarity was quantified in two ways. First, five independent judges were asked to inspect visually sound-spectrogram of early subsong bouts and food begging calls of juvenile males (n = 5) and agreed that approximately 10–33% of early subsong was very similar to the late begging calls of males at PHD25, but not to those of female calls or to the begging calls of younger males (Fig. S3). Second, we used similarity scores from Sound Analysis Pro [25] to compare each male's begging calls and early subsong. Approximately 7–38% of the total duration of the sounds of subsong (range of all males) resembled that same male's begging calls at PHD25 with a similarity score of 67–85. None of the early subsong bouts matched male calls at PHD15 or female calls at PHD25 (Fig. S3). The “begging call-like” subsong matched late begging calls in almost all sound features (MANOVA, Wilk's Lamda = 0.086, P>0.05; Tukey post-hoc test) except the lower amplitude in subsong (Kolmogorov-Smirnov test, z = 4.18, P<0.001). The incidence of “begging call-like” sounds in subsong gradually subsided in the next few weeks. Females do not sing as adults and have no subsong.

### Deafening experiments

**Figure 3:**
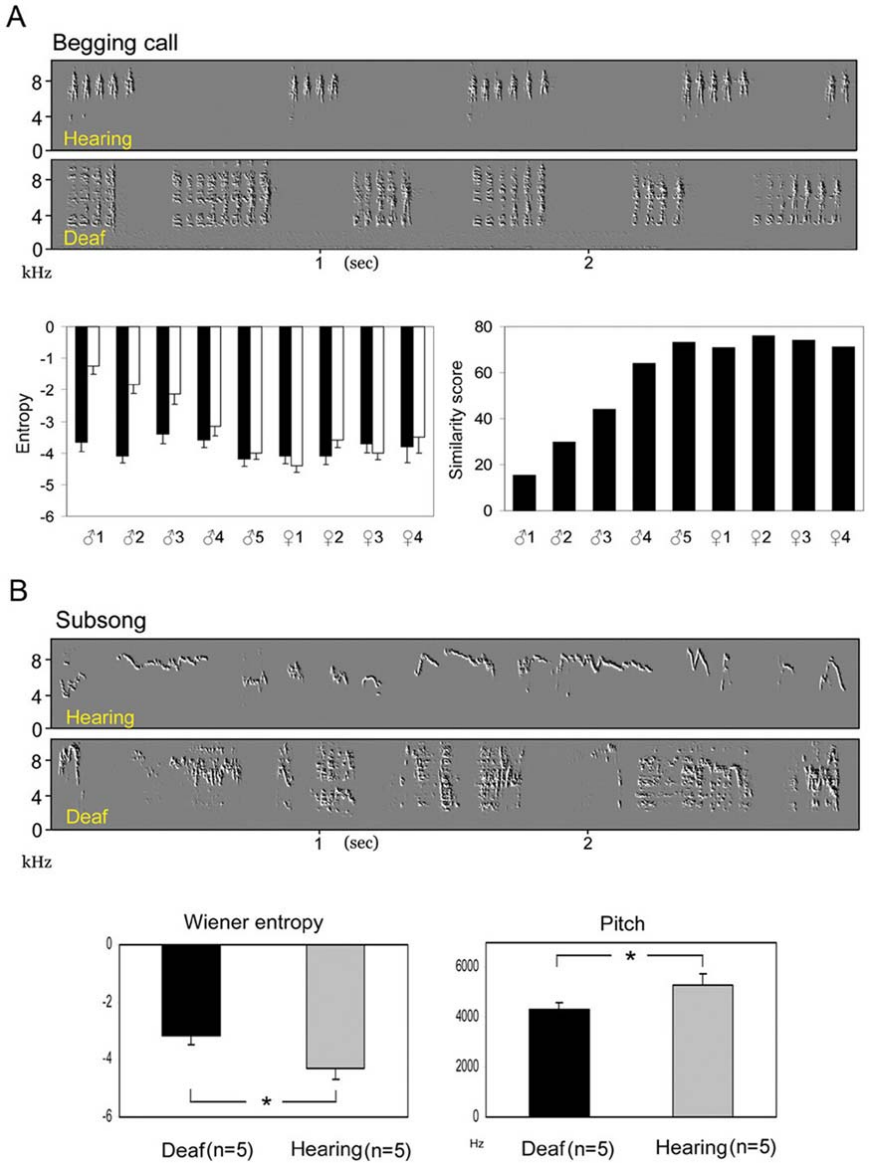
The effect of hearing on food begging calls and subsong. (A) On the lower left panel, after deafening, the food begging calls of males (n = 5) had significantly higher entropy (white bars, 300 notes per bird; two-sample Kolmogov-Simorov test, z = 2.14, P<0.01) than the entropy of pre-operative birds (black bars), where the first three males had largest increase in entropy after deafening. On the lower right panel the first three males also showed lower similarity scores when comparing their pre- and post-deafening food begging sounds. (B) The subsong of deaf males (n = 5) was significantly different from that of intact-hearing males (n = 5) with higher entropy (lower left panel; 300 notes per bird; Kolmogorov-Smirnov test, z = 2.32, P<0.001) and lower pitch (lower right panel; z = 2.84; P<0.001).

We then tested whether begging calls required auditory feedback by deafening young males (n = 5) and females (n = 4) at PHD18–28, before subsong was produced. One to three days after deafening, the begging calls significantly changed in three of the deaf males, whose calls had significantly higher entropy and lower pitch than preoperatively (Fig. 3 and S4; Audios S5, S6). The begging calls of the equally aged sham-control males or unilaterally deafened males did not changed significantly (n = 8; z = 1.6–2.1, P>0.05). The “chip” contact call of males was not altered by deafening (MANOVA,Wilk's Lamda = 0.24; F = 58.1; P>0.1). The food begging calls were not significantly affected by deafening in the deaf females (P>0.05; Fig. S4).

The early subsong of deaf males (n = 5; PHD 38–45), as a group, was significantly different from that of hearing controls (n = 5; PHD 40–45), with higher entropy and an absence of pure high pitched whistles (Figs. 3, S5; Audios S7, S8). The subsong of one of the birds did not change significantly after deafening (MANOVA, P>0.05). Our previous study [26] showed that the plastic song and adult song developed by early deafened sparrows were also significantly different from those of their hearing controls. However, the extent of the effect of early deafening on begging calls, subsong, plastic song, or adult song varied between individuals.

### *C-fos* expression

**Figure 4:**
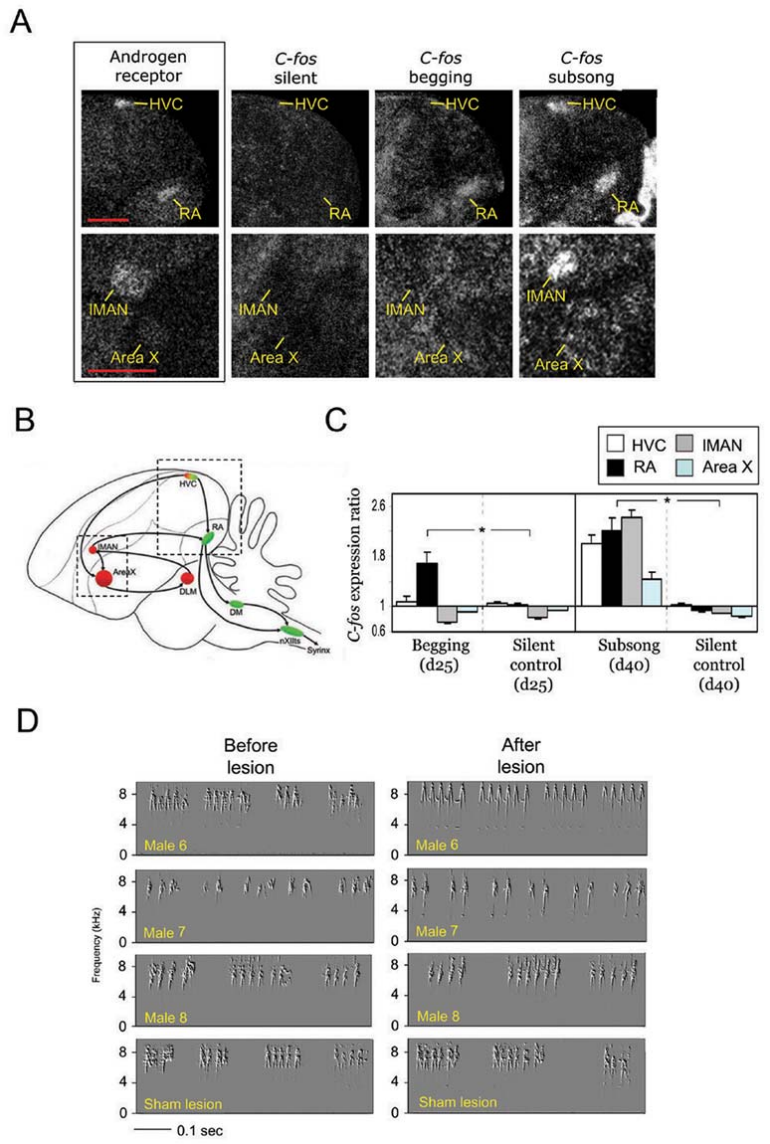
The involvement of the forebrain's nucleus RA during the food begging behavior. (A) Food begging calls induced the expression of immediate early gene *c-fos* only in RA but not HVC, lMAN, or AreaX. Subsong induced *c-fos* expression in all four song nuclei. Four telencephalic song system nuclei can be identified by using androgen receptor as a marker. Calibration bar  = 1 mm. (B) Saggital view of the forebrain song system. (C) *C-fos* expression ratio (song nucleus/surrounding regions) was significantly different in nucleus RA, but not in three other song nuclei, between begging males and non-begging controls (left panel of 4C; Friedman two-way ANOVA; *X* ^2^ = 25.1; P<0.02); the c-fos expression ratios was higher in all 4 major song nuclei of the birds producing subsong than in birds there were silent (right panel of 4C; Friedman two-way ANOVA; *X* ^2^ = 37.9; P<0.01). (D) Electrolytic lesion of RA reduced acoustic variability of the food begging calls. The food begging calls of 3 juveniles males before and after electrolytic lesion of nucleus RA. Male 8 is a sham-control male.

We tested whether the production of begging calls was associated with the forebrain song circuits by using an immediate early gene, *c-fos*, as a neural activity marker [27]. It is known that singing in songbirds induces *c-fos* expression in forebrain song nuclei RA, HVC, Area X, and LMAN [28], [29]. Intense food begging for 30 minutes in male sparrows (n = 6) at PHD20–25 induced significantly higher levels of c-*fos* expression in one of the forebrain nuclei, RA (Fig. 4B), than in non-begging birds (n = 3; juveniles that were silent but could hear the begging calls of others). *C-fos* expression was significantly higher in RA than in surrounding arcopallium in begging males (n = 6) but no such difference was observed in non-begging controls (n = 3) (Mann-Whitney U Test, W = 35, P<0.01; Fig. 4C). There was no increase in the level of *c-fos* expression in RA of begging females of the same age (n = 3; Mann-Whitney U Test, P>0.05). No significant difference was found in HVC, lMAN, and Area X in begging vs. non-begging control birds (Fig. 4C). Production of the contact call did not induce significant *c-fos* expression in RA (n = 3; Mann-Whitney U Test, W = 19, P>0.05; two-tails). However, *c-fos* was highl y expressed in all four major forebrain song nuclei of juveniles producing early subsong (n = 4 males), with no such expression in silent birds (n = 3).

### Electrolytic lesion

The *c-fos* induction in the premotor nucleus RA during production of food begging suggests a possible involvement of forebrain song circuitry. To test this idea, juveniles (n = 4 males and 3 females) at PHD 21–22 received complete bilateral lesions of RA. After 1–2 days, the food begging calls of post-operative males were significantly different from those produced before lesions (n = 4 males, 300 notes each; MANOVA test of 6 sound features; Wilk's Lamda = 0.83, F = 77.5, P<0.01; Fig.4D, Audios S9, S10). No significant acoustic change was found in the begging calls of the control males (MANOVA, Wilk's Lamda = 0.27; F = 46.3; P>0.1; Fig. 4D) or in the “chip” contact calls of males (MANOVA, F = 38.4; P>0.1).

## Discussion

Our results suggest that the food begging calls of male chipping sparrows show characteristics that are associated with vocal learning. The acoustic structure of the food begging calls varies among individuals and changes with age. Early deafening and bilateral lesions of the forebrain song control nucleus RA affected the food begging calls of some male, but not female, fledglings. In addition, the production, but not hearing, of begging calls induced *c-fos* expression in the nucleus RA of males; there was no noticeable rise in *c-fos* expression in other song nuclei of the male forebrain or in the RA of females. The subsequent incorporation of food begging calls into subsong leaves open the possibility that vocal experience that might have been gleaned during the earliest stage is then incorporated into subsong, all this happening well before imitation of external models gets started. While the auditory-sensitive food begging calls and subsong are reminiscent of what Marler et al. [9] called “improvisation”, they are not, by themselves, evidence of learning. We do not claim that the food begging calls of male chipping sparrows are learned, but that they are at the beginning of a process that leads to vocal learning. Regardless what we choose to call this early effect of hearing on vocal ontogeny, it seems clear that a self-centered effect of hearing on vocal output precedes an effect of hearing that relies on imitation of external models. Proof of learning, in the form of imitation, comes later in ontogeny (plastic song stage).

The extent to which intact hearing contributed to vocal ontogeny differed between individuals. In our study, early deafening changed the begging calls of 3 out of 5 males but did not affect the begging calls of the other two, whose calls were as stereotyped as those of females. Interestingly, one of these birds also produced normal subsong. Marler and Sherman [30] had already noticed that even in early deafened songbirds, comparisons between species revealed differences in their aberrant, adult song. These differences emerged because the song of the deaf birds, despite its many abnormalities, preserved some species-specific features. These authors concluded that birds that learned their song built their skill around innate perceptual and motor predispositions, so that learning added to innate programs and did not start from a *tabula rasa*. Our observations on male chipping sparrows suggest that the extent of dependence on these three sources of information – innate motor, innate perceptual and learned by reference to auditory information – differs even among individuals of a same species and moreover that this ratio can change as vocal development progresses. It is unlikely that the effect of deafening on the food begging calls of 3 out of 5 males was a fluke, for the effect on those 3 males was very robust. Importantly, this effect was absent from all 4 deafened females and from 8 control or unilaterally deafened males. In addition, as noted above, the early effects of hearing on vocal ontogeny are part of a cluster of features associated with vocal learning that occurs in male, but not female chipping sparrows.

Alternatively, the different effect of deafening on call variability could be due to differentiation of vocal-motor program that enables and guides song learning. Hearing and vocal output might interact in a chain of input-output events, as is the possibility that hearing might act directly on the development of vocal-motor pathways. This differentiation may involve auditory sensitivity in forebrain nucleus RA. If such sensory-sensitive differentiation occurs early in development, this might explain why there is no significant deafening effect on some of the males. Moreover, as described in Introduction, there are various ways whereby hearing could modify vocal output, though at present study we do not know in which of these ways hearing affected the begging calls of male chipping sparrow fledglings. It would be interesting to compare the morphology of RA in male fledglings whose begging calls were or were not affected by deafening.

Given the male/female differences in vocal ontogeny, which of the two is primitive? Instances of sexual dimorphism in the song system highlight nuclei and pathways specialized for vocal learning that are often much more developed in males than in females, particularly in temperate zone species [31]. Chipping sparrows conform with this pattern, and so we might infer that the vocal ontogeny of females is closer to the primitive condition than that of males. If so, then the innate motor programming that is responsible for the production of stereotyped food begging calls in female juveniles may be close to what existed before hearing commenced to have an effect on vocal ontogeny. RA lesions completely abolish the production of subsong and adult song in male zebra finches [32]. The fact that RA lesions in male chipping sparrows do not abolish begging calls, but rather reduce call variability, suggests that the basic program for begging calls in males and females is represented at midbrain vocal centers, and that in males the descending input from RA introduces variability. The source of this variability remains unknown, but apparently can be influenced by hearing. The variability expressed in the male begging calls could arise within RA or be driven from lMAN, that projects to RA. Several studies have shown that in zebra finches LMAN is a generator of vocal variability [33], [34]. Though *c-fos* expression in LMAN did not increase during food begging behavior in our chipping sparrows, early lMAN activity may have been too weak to induce in it a noticeable rise in *c-fos* expression.

Intriguingly, the “chip” contact call that develops at about the same age as begging calls of fledglings was not affected by deafening or electrolytic lesion of song nucleus RA, nor was its production associated with *c-fos* expression in RA. Perhaps early in ontogeny, there are two circuits that generate vocal signals: one is not sensitive to auditory feedback and is not dependent on RA; and a second one is auditorily guided and dependent on RA. A similar dichotomy has been described in learned and non-learned calls of zebra finches [22].

Our observations on chipping sparrow suggest that a self-centered influence of hearing on vocal ontogeny, during food begging calls and subsong, precedes hearing-dependent imitation. The variability and auditory sensitivity of the begging calls of males could be due to the differentiation of vocal-motor program that enables and guides song learning. This precedence may apply not just to the behavior, but also to the circuitry required for either type of hearing-dependent vocal ontogeny. Our observations suggest that the self-centered ontogeny requires fewer relay stations, and these closer to the brain stem, than is the case for pathways associated with vocal imitation (Fig. S6). We view our results as a first probe into just how a vocal learning system puts itself together, both during ontogeny and in evolutionary time. Other approaches and more comparative work will be needed to test our inferences and to produce alternative models for the ontogeny and evolution of vocal learning. We do not claim that the food begging calls in chipping sparrows are learned, but that they are at the beginning of a process that leads to vocal learning.

This longitudinal, ontogenetic look at how vocal learning emerges in the individual chipping sparrow may be of use for trying to understand how vocal learning evolved. As in chipping sparrows, the pre-speech sounds of infants show acoustic continuum between the sounds of crying, babbling, and early speech [35], and all these sounds are different between hearing and deaf infants [36]–[38]. In songbirds and humans the earliest vocalizations may already be part of a vocal learning program that culminates in the imitation of external sounds.

## Materials and Methods

### 1. Experimental subjects

We chose a seasonal songbird, the chipping sparrow as the experimental subject. Only male chipping sparrows sing and each adult male has only one single song type, which consists of repetitions of the same syllable. This very simple song is acquired by precise imitation from an adult neighboring male [39]. The entire developmental program, from subsong to full song, lasts 8–10 months. The simple, easily quantifiable song repertoire of male chipping sparrows and the well-studied natural history of the phenomenon [39] provide convenient material to search for the earliest evidence of vocal learning.

Nestling chipping sparrows (n = 68) were collected at post-hatching days (PHD) 3–7 from nests in the wild at the Rockefeller University Field Research Center in Millbrook, New York. Juveniles were hand reared until independence (at PHD 30–36) feeding them a modified Lanyon diet [40] plus mealworms and wax worms. Some of these birds were repeatedly used for two or more experiments. The parent birds were not collected. The sex of each individual bird was first determined from blood samples using PCR amplification of CHD gene fragments following the protocol of Griffith et al [41] and the sex was later confirmed when the birds were sacrificed and their gonads examined. Animal protocol was reviewed and approved as meeting appropriate ethical standards by The Rockefeller University's IACUC boards.

### 2. Sound recording and analysis

Juveniles were housed singly in a sound-proof chamber. The door to the chamber was open so that each bird (n = 13 males and 12 females) could hear or see other birds housed in the same room. This social setting was required because if the door to the chamber were kept closed the juveniles stopped begging. Even with the door open, this setting attenuated other sounds, allowing for good recordings of the bird's vocalizations. The food begging calls were defined as the vocalizations produced by a juvenile as the food was presented a few inches in front of it after a fasting of approximately 1 hour. Begging calls were recorded 2–6 times per day, with at least one recording session in the early-morning (0600–0800) and one in the late afternoon (1600–1800). For sound recording, we used an Audio technica AT803 Lavalier microphone (Audio-Technica U.S., Inc. Stow, Ohio) that was placed in the top center of the cage and was connected to an M-audio Audio-Buddy pre-amp (Avid Technology, Irwindale, CA), an M-audio Delta 44 sound card and to Sound Analysis Pro (SAP) software, version 1.04 (with default setting). During each recording session, a small amount of food was slowly moved towards the bird until 2–3 minutes of calls were recorded; and approximately 300–800 call notes were recorded per bird each day. Subsong and other vocalizations were continuously recorded until 2 months of age. We manually adjusted the gain level of pre-amplifier to record the low amplitude subsong.

#### Sound analysis

Quantitative begging-call and subsong analysis was performed using Sound Analysis Pro program (SAP). Each bird's food begging calls and subsong were analyzed at the level of a single note (a call note was defined as a continuous sound preceded and followed by silent intervals of >5 ms) or a rendition (delivered in a quick succession of repeated notes, Fig. 1). Quantification of the acoustic properties of food begging calls and/or subsong and comparisons between age/sex/treatment groups was done using a similarity score obtained from the SAP for asymmetric pairwise comparisons. The frequency range was adjusted to 11800 Hz in the setting. The sound intervals (9.27 ms) used for such comparison were characterized by measures from 6 acoustic features: duration, pitch, frequency modulation (FM), Wiener entropy, mean frequency, and pitch goodness (PG). SAP calculates the Euclidean distance between all interval pairs from two notes over the course of the begging calls. To determine whether or not the begging call structure was significantly different between sexes or changed with age, we analyzed each bird's begging calls at two developmental ages (PHD 15–16 and PHD 25–26). Each bird's calls were compared using the 6 call parameters listed above and multivariate analysis of variance, MANOVA (SPSS 16.0), to determine whether the variability of sound features between the calls from two groups of birds of different age or sex, for example, were significantly different from each other. Wilk's lambda and overall F value were used to test for significance, with Tukey post-hoc test for each variable. In addition, we used two-sample Kolmogorov-Smirnov test with Bonferroni correction to test the significant difference in each of six acoustic features between sexes.

To quantify the similarity between the food begging calls and subsong, we collected the recordings of the first 30 subsong bouts produced by each juvenile (each bout lasted from 4∼10 s and was preceded and followed by a silent interval >2 s) during the first 2–3 days starting at about PHD 33–42. For the comparisons with subsong we chose the same male's begging calls recorded at PHD 15–16 and 25–26 and female calls recorded at PHD 25–26 (about 25 food begging renditions per bird at each age or sex group). Two different approaches were used to compare the similarity between subsong and food begging calls: 1) Visual inspection: five judges compared the spectrogram printouts from early subsong (defined as subsong recorded during the first 2–3 days of subsong production), using 30 subsong bouts from each bird and food begging calls from the same birds at PHD 15, PHD 25 and females at PHD 25. The judges did not know the sex or age of the individuals. Judges were asked to assign a score from 0 (no similarity) to 5 (very similar) to each comparison. 2) Similarity measurement: we used the similarity score from SAP and used each subsong session of a male to match each of all the begging call renditions. For this comparison, each subsong bout was manually segmented into 300 ms “rendition”. Each rendition was then automatically compared with begging call bouts (n = 25) of similar duration using the batch function of SAP. The highest score of all these comparisons was selected to determine the number and proportion of subsong renditions that best matched the begging calls. The proportion of begging call-like subsong was calculated by the total duration of the begging call-like sounds, determined by high similarity score, divided by the total duration of the subsong. The two-sampled Kolmogorov-Smirnov test was used to test for significant differences in univariate distribution of begging call and subsong features.

### 3. Deafening

Juvenile chipping sparrows of both sexes (n = 5 males and 4 females) were deafened at PHD 18–28 by bilateral removal of both cochleae. Each bird was anesthetized with 0.07–0.08 ml of 1∶5 Nembutal. The tympanic membrane and the columella were removed, and a fine wire hook was inserted through the oval window to engage and then pull out the cochlea. The tympanic membrane then grows back. Eight other birds were used as controls (three with removal of just one cochlea and five intact). Before surgery, each experimental bird was housed singly in a recording chamber and the begging calls were recorded for five days. Soon after recovery from surgery, the operated birds were placed back in the same recording chamber and their vocalizations (begging calls and subsong) were immediately recorded until two months of age. We used the same five deaf males and intact controls to test the effect of early deafening on subsong. The two-sample Kolmogorov-Smirnov test was used to test for significance of differences in univariate (call or subsong) feature distribution comparing pre-operative and post-operative birds or comparing deaf birds and hearing controls.

### 4. *In situ* hybridization

Juvenile sparrows (n = 6 males) were sacrificed after producing 30 minutes of food begging calls (2–5 minutes of food-begging followed by 5 min. of silence and so on) in the early morning, which were recorded using the Raven 1.2 (Cornell laboratory of Ornithology, Ithaca, New York) program. Approximately15–20 min after the end of begging the birds were decapitated. Brains were removed and stored in −80°C. Three juvenile males that were prevented from begging (i.e., hand-feeder was present but not close to the birds) were used as controls. The non-begging birds did produce many contact calls and they were able to hear the begging calls of other juveniles. The contact calls were induced by the presence of a hand-feeder who was about 10 feet away, approximately 150–400 contact calls were recorded from each bird during a 30-min period. We counted the number of calls produced by each bird by examining the spectrograms from our continuous recordings. For subsong, juveniles (n = 4 singing males) were sacrificed after 30–40 minutes of subsong singing in the morning. Three silent males were the controls.

*In situ* hybridizations were performed and quantified following a protocol described previously [42]–[43] using ^33^P-labeled riboprobes. After the bird was sacrificed, the brain was removed and sectioned by cryostat. In brief, frozen brain sections (10 um) were hybridized with ^33^P-labeled antisense c-fos riboprobes and the sections were overlaid by X-ray film for a few days. After developing the X-ray films, the brain image on the exposed film was placed under a dissecting scope (Leica, W340) and captured by the computer using a Spot IV camera and Spot software 3.2.4 (Diagnostic Instruments, Sterling Heights, MI). Images were transferred to Photoshop (Adobe, San Jose, CA) and converted to gray scale. Vocal nuclei and adjacent non-vocal areas were outlined and the average pixel density was calculated using the Photoshop histogram function. C-fos expression was quantified in several nuclei and their adjacent non-vocal areas in this manner, e.g., the caudal nidopallium under HVC; nonauditory arcopallium next to the robust nucleus of the arcopallium (RA); nidopallium rostral to lateral magnocellular nucleus of the anterior nidopallium (lMAN); caudal striatum immediately caudal to Area X. To calculate ratios of differential expression in vocal nuclei relative to their surrounding brain subdivision, the pixel density of a song nucleus was divided by the pixel density of the respective adjacent region with comparable size for quantification. Freedman two-way ANOVA and Mann-Whitney two-tailed U test was used to determine if the gene expression ratio of begging males was significantly different from that of the non-begging control birds or begging females. The androgen receptor gene was used as marker to identify the four forebrain song nuclei, HVC, RA, lMAN, and AreaX.

### 5. Electrolytic lesion

Juvenile sparrows (n = 4 males and 3 females) received complete bilateral lesions of nucleus RA. We used size 000 insect pins (Carolina Biologicals) insulated with Insl-x (Insl-X Product Corp.) as electrodes. A single penetration per RA delivering 50 uA for 40 sec was sufficient. For the control group (n = 3 males), the lesion was done by a single penetration in the arcopallium outside and next to RA. Each of the 9 pre-operative males was placed in a sound-proof chamber and its begging calls and contact calls were recorded for at least 3 consecutive days immediately prior to surgery. After recovering from surgery, the operated birds went back to the sound-proof chamber. The begging calls, contact calls, and other sounds were recorded continuously for 3–5 days. To identify the effectiveness of lesions targeted at RA, birds were perfused under anesthesia (Nembutal) with PBS followed by 4% paraformaldehyde. Brains were then removed and sectioned (50 um) in a vibratome (Lancer). All sections were stained with a 0.3% solution of cresyl violet acetate (Sigma). We identified any remaining RA cells by their relatively larger size and estimated the amount of RA tissue remaining after lesions, expressed as a percentage of the mean volume of RA in the intact controls. The Kolmogorov-Smirnov two- sample test was used to test for significance when comparing begging call or contact call features before and after RA or sham-lesions.

## Supporting Information

Figure S1 The vocal ontogeny of a male chipping sparrow. Chipping sparrows are seasonal songbirds, the adult song does not fully develop until 8–10 months of age. The earliest vocalizations of chipping sparrows are the food begging calls that start as high-pitched pure tones at 3–4 days after hatching (d4). These calls gradually become segmented with sharper frequency modulation. The late begging calls (d25) closely resemble some sounds of early subsong (d40). During the plastic song stage (d250), as shown in previous study (6), the male sparrows develop several “precursor” song types, only one of which (yellow dot) is modified to match the tutor song and then crystallized as adult song.
(7.30 MB TIF)

Figure S2 Sexual dimorphism of food begging calls at PHD 20 as revealed by 6 acoustic features: duration, pitch, frequency modulation (FM), Wiener Entropy, pitch goodness, and mean frequency. All of six features differed significantly between the sexes (two-sampled Kolmogorov-Smironov test with Bonferroni correction, P<0.001).
(6.96 MB TIF)

Figure S3 Quantitative measures between food begging calls and early subsong. (A) Five independent judges used spectrogram printouts of subsong renditions to compare with female calls at PHD25, male calls at PHD15 and PHD25. The judge did not know the sexes and age of each call rendition. Judges were asked to assign a score from 0 (no similarity) to 5 (very similar) to each comparison. The judges agreed that a small portion of subsong best matched the begging calls of males at PHD25. (B) We used similarity score from Sound Analysis Pro to compare early subsong and food begging calls. Approximately 7–38% of subsong resembled PHD25 male calls. Female calls and the calls of younger males at PHD15 did not match any of the subsong sessions.
(5.43 MB TIF)

Figure S4 Deafening effect on the food begging calls. (A) after deafening, the food begging calls of juvenile males (Males 1–3) significantly changed with higher entropy and lower pitch. The food begging calls of 4 females did not change after deafening (B). (C) The contact calls of a juvenile male before and after deafening.
(8.32 MB TIF)

Figure S5 Deafening effect on subsong. The early subsong bout of a deaf male at PHD 40 was significantly different from that of a hearing control at the same age, with higher entropy and an absence of high pitched pure-tone whistles.
(8.41 MB TIF)

Figure S6 The early stage of vocal learning for food begging is, behaviorally and circuit wise, a simpler phenomenon that precedes and leads to the development and evolution of vocal imitation. The male begging calls are affected by deafening, and a forebrain premotor nucleus RA is involved in call production. By contrast, the innate “chip” contact calls developed in fledgling sparrows are not affected by deafening nor is nucleus RA involved in their production. The development of normal subsong, plastic song, and adult song in chipping sparrows requires auditory feedback and their production engages all of the song system nuclei shown.
(7.62 MB TIF)

Movie S1 The food begging calls of nestlings (8 day old)
(3.37 MB MOV)

Movie S2 The food begging calls of fledglings (23 day old)
(4.95 MB MOV)

Movie S3 Subsong singing of a juvenile male (38 day old)
(5.29 MB MOV)

Audio S1 The food begging calls of a female sparrow at PHD20
(0.21 MB MP3)

Audio S2 The food begging calls of a male sparrow at PHD20
(0.26 MB MP3)

Audio S3 The food begging calls of a male sparrow at PHD26
(0.17 MB MP3)

Audio S4 The early subsong of a male sparrow at PHD39
(0.38 MB MP3)

Audio S5 The food begging calls of a male WP before deafening
(0.25 MB MP3)

Audio S6 The food begging calls of a male WP after deafening
(0.25 MB MP3)

Audio S7 The subsong of an intact hearing sparrow
(0.24 MB MP3)

Audio S8 The subsong of a deaf sparrow
(0.25 MB MP3)

Audio S9 The food begging calls of a male sparrow LBY at PHD25 before RA lesion
(0.08 MB MP3)

Audio S10 The food begging calls of a male sparrow LBY 2-day after RAlesion
(0.08 MB MP3)
