# Supplementary figures and images for: Variable Food Begging Calls Are Harbingers of Vocal Learning
Source: PLoS One. 2009 Jun 16;4(6):e5929. doi: 10.1371/journal.pone.0005929 (PMC2691483; doi:10.1371/journal.pone.0005929)

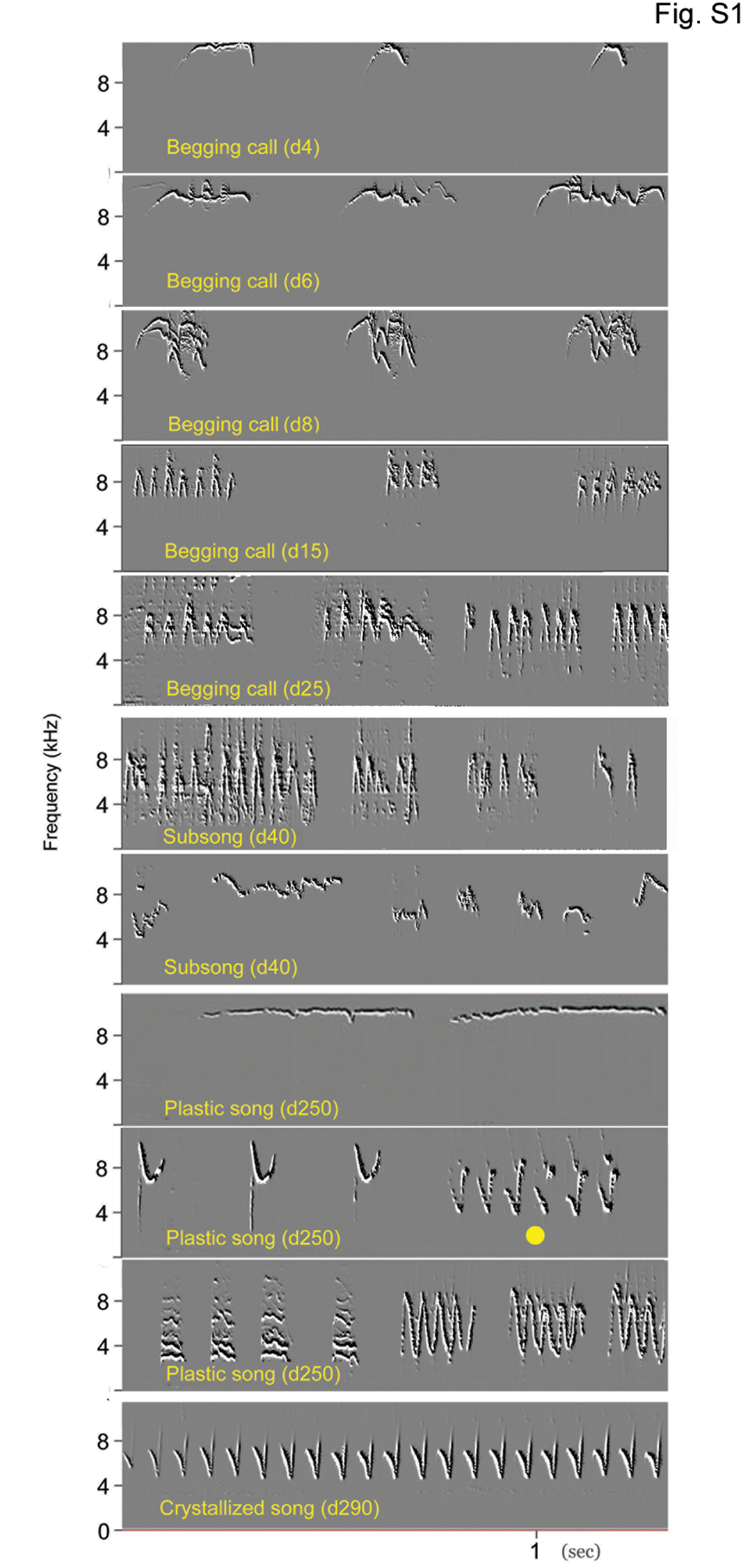

Supplement: Figure S1 — The vocal ontogeny of a male chipping sparrow. Chipping sparrows are seasonal songbirds, the adult song does not fully develop until 8–10 months of age. The earliest vocalizations of chipping sparrows are the food begging calls that start as high-pitched pure tones at 3–4 days after hatching (d4). These calls gradually become segmented with sharper frequency modulation. The late begging calls (d25) closely resemble some sounds of early subsong (d40). During the plastic song stage (d250), as shown in previous study (6), the male sparrows develop several “precursor” song types, only one of which (yellow dot) is modified to match the tutor song and then crystallized as adult song. (7.30 MB TIF) [file pone.0005929.s001.tif]

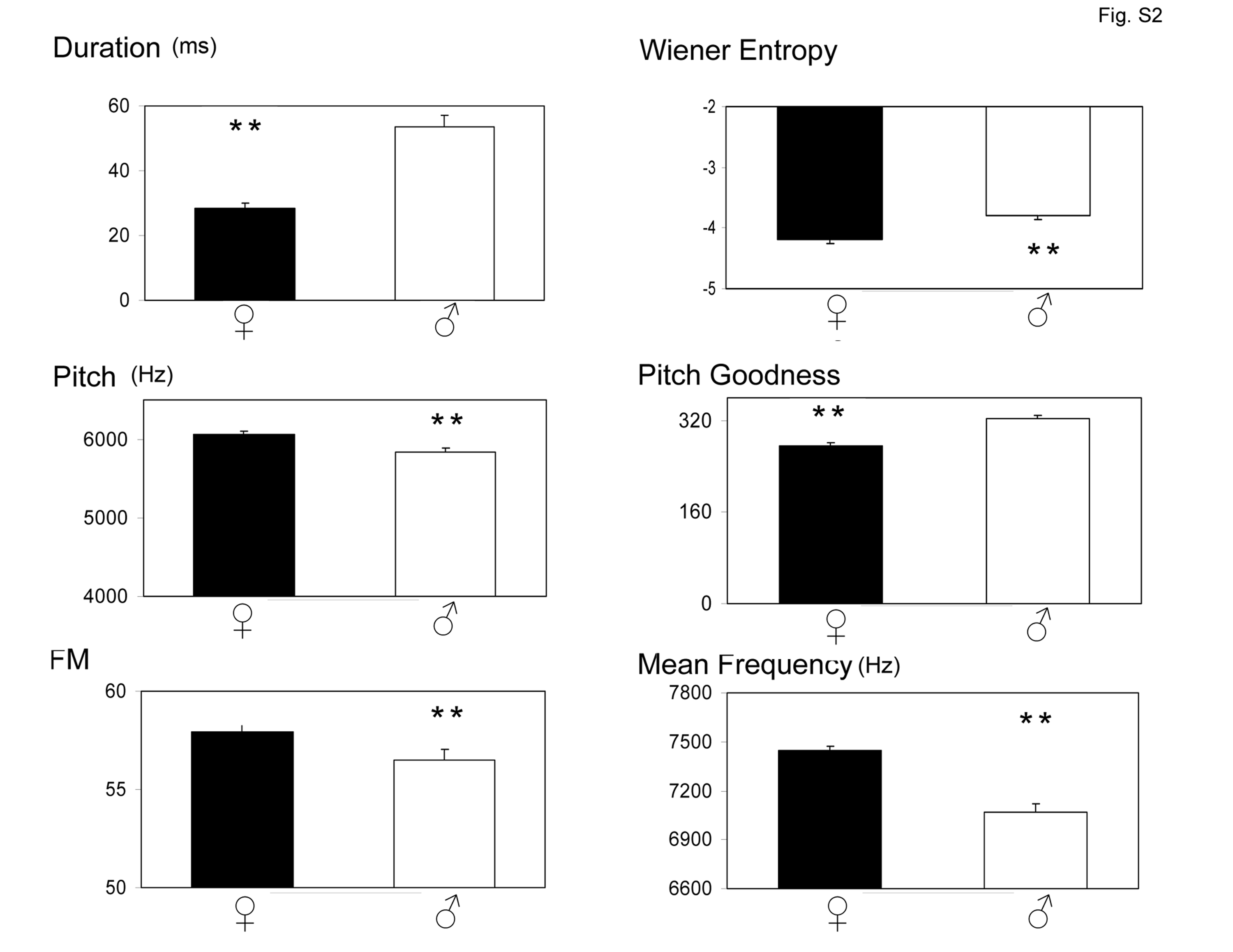

Supplement: Figure S2 — Sexual dimorphism of food begging calls at PHD 20 as revealed by 6 acoustic features: duration, pitch, frequency modulation (FM), Wiener Entropy, pitch goodness, and mean frequency. All of six features differed significantly between the sexes (two-sampled Kolmogorov-Smironov test with Bonferroni correction, P<0.001). (6.96 MB TIF) [file pone.0005929.s002.tif]

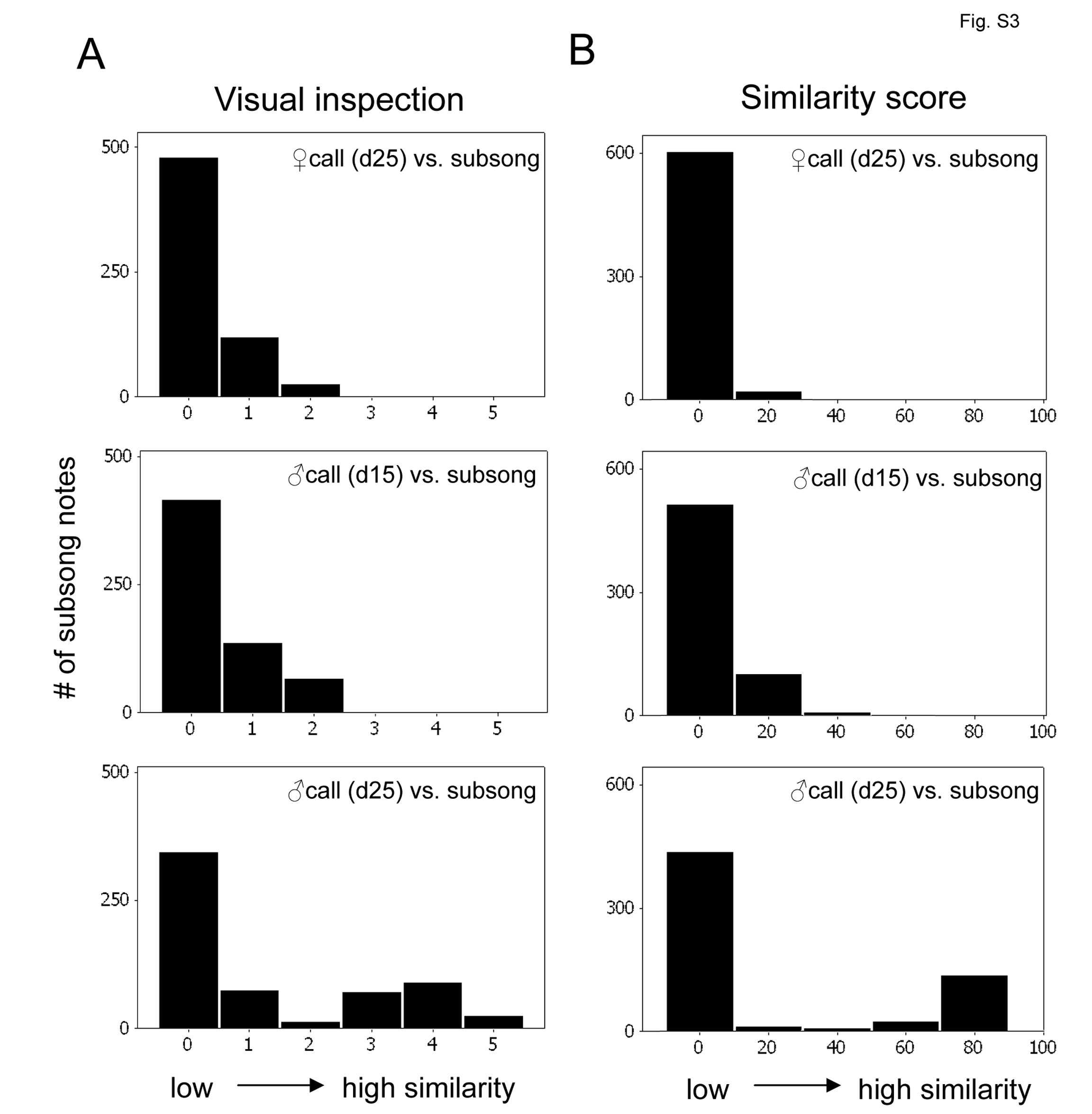

Supplement: Figure S3 — Quantitative measures between food begging calls and early subsong. (A) Five independent judges used spectrogram printouts of subsong renditions to compare with female calls at PHD25, male calls at PHD15 and PHD25. The judge did not know the sexes and age of each call rendition. Judges were asked to assign a score from 0 (no similarity) to 5 (very similar) to each comparison. The judges agreed that a small portion of subsong best matched the begging calls of males at PHD25. (B) We used similarity score from Sound Analysis Pro to compare early subsong and food begging calls. Approximately 7–38% of subsong resembled PHD25 male calls. Female calls and the calls of younger males at PHD15 did not match any of the subsong sessions. (5.43 MB TIF) [file pone.0005929.s003.tif]

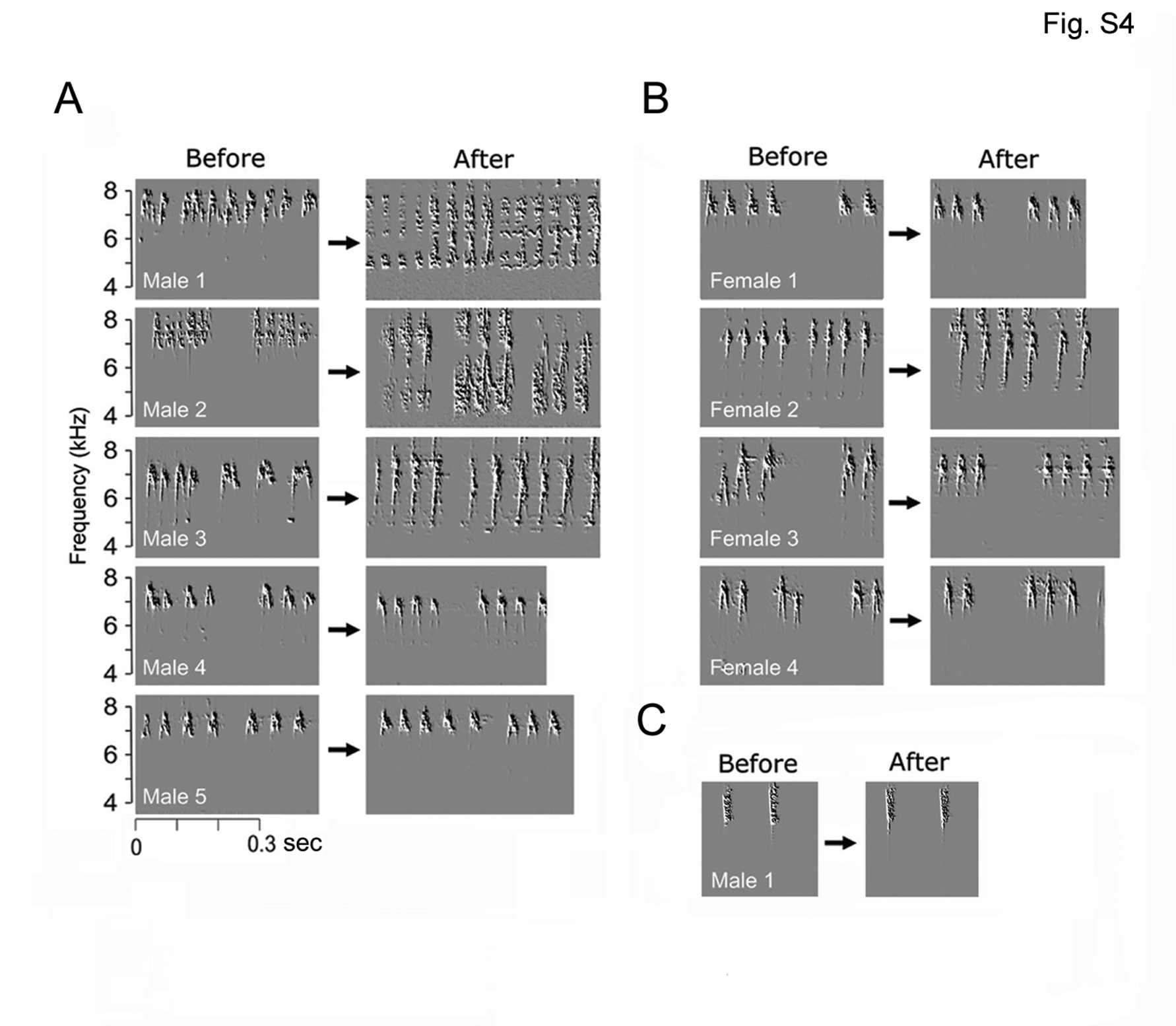

Supplement: Figure S4 — Deafening effect on the food begging calls. (A) after deafening, the food begging calls of juvenile males (Males 1–3) significantly changed with higher entropy and lower pitch. The food begging calls of 4 females did not change after deafening (B). (C) The contact calls of a juvenile male before and after deafening. (8.32 MB TIF) [file pone.0005929.s004.tif]

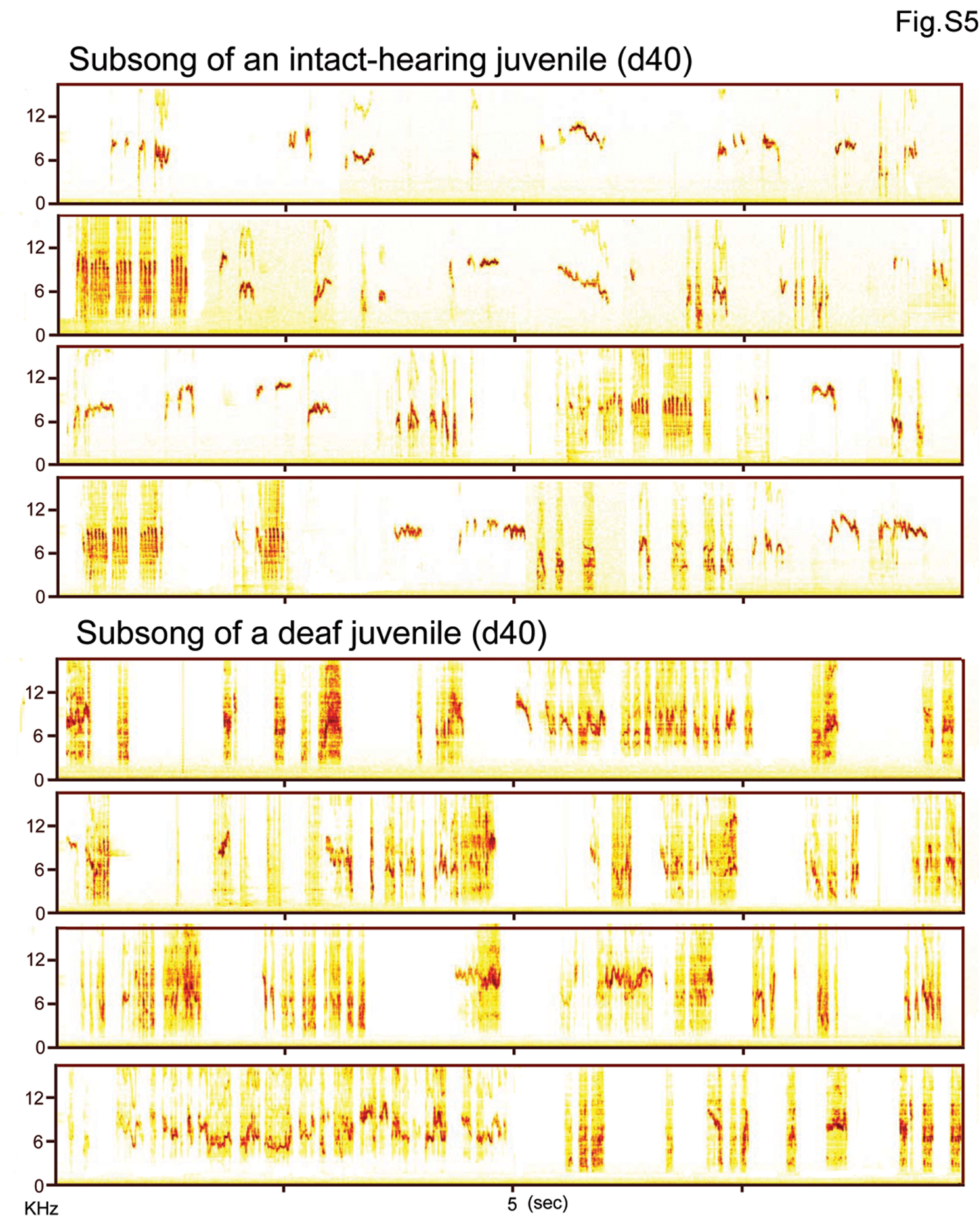

Supplement: Figure S5 — Deafening effect on subsong. The early subsong bout of a deaf male at PHD 40 was significantly different from that of a hearing control at the same age, with higher entropy and an absence of high pitched pure-tone whistles. (8.41 MB TIF) [file pone.0005929.s005.tif]

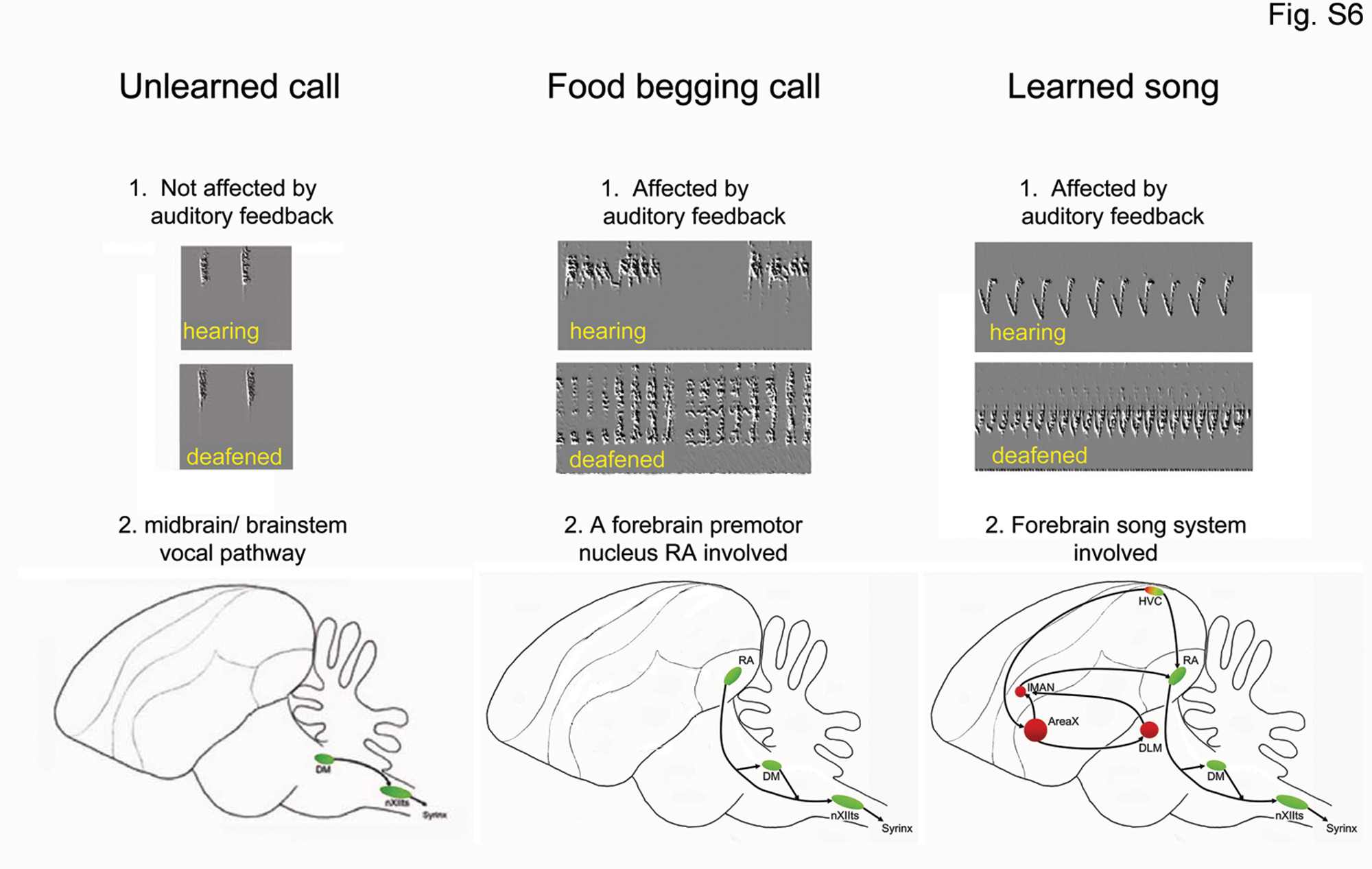

Supplement: Figure S6 — The early stage of vocal learning for food begging is, behaviorally and circuit wise, a simpler phenomenon that precedes and leads to the development and evolution of vocal imitation. The male begging calls are affected by deafening, and a forebrain premotor nucleus RA is involved in call production. By contrast, the innate “chip” contact calls developed in fledgling sparrows are not affected by deafening nor is nucleus RA involved in their production. The development of normal subsong, plastic song, and adult song in chipping sparrows requires auditory feedback and their production engages all of the song system nuclei shown. (7.62 MB TIF) [file pone.0005929.s006.tif]
